# Anthropometric Determinants of Respiratory Sinus Arrhythmia in Children

**DOI:** 10.3390/ijerph19010566

**Published:** 2022-01-05

**Authors:** Paulina Lubocka, Robert Sabiniewicz, Klaudia Suligowska, Tomasz Zdrojewski

**Affiliations:** 1 Department of Pediatric Cardiology and Congenital Heart Disease, Medical University of Gdańsk, Dębinki Street 7, 80-952 Gdańsk, Poland; sabini@gumed.edu.pl; 2 Department of Preventive Medicine and Education, Medical University of Gdańsk, Dębinki Street 7, 80-952 Gdańsk, Poland; klaudia.suligowska@gumed.edu.pl (K.S.); tz@gumed.edu.pl (T.Z.); 3 Department of Dental Techniques and Masticatory System Dysfunctions, Medical University of Gdańsk, Tuwima Street 15, 80-210 Gdańsk, Poland

**Keywords:** cardiology, human development, electrocardiography, physiology

## Abstract

Background: The study was conducted to investigate the implications of anthropometry in school-aged children on the degree of respiratory sinus arrhythmia observed in clinical settings. Methods: In a cohort study, 626 healthy children (52% male) aged 10.8 ± 0.5 years attending primary school in a single town underwent a 12-lead electrocardiogram coupled with measurements of height, weight and blood pressure. Indices of respiratory sinus arrhythmia (pvRSA, RMSSD, RMSSDc) were derived from semi-automatic measurements of RR intervals. Height, weight, BMI, blood pressure as well as waist and hip circumferences were compared between subjects with rhythmic heart rate and respiratory sinus arrhythmia, and correlations between indices of sinus arrhythmia and anthropometry were investigated. Results: Respiratory sinus arrhythmia was recognized in 43% of the participants. Subjects with sinus arrhythmia had lower heart rate (*p* < 0.001), weight (*p* = 0.009), BMI (*p* = 0.005) and systolic (*p* = 0.018) and diastolic (*p* = 0.004) blood pressure. There were important inverse correlations of heart rate and indices of sinus arrhythmia (r = −0.52 for pvRSA and r = −0.58 for RMSSD), but not the anthropometry. Conclusion: Lower prevalence of respiratory sinus arrhythmia among children with overweight and obesity is a result of higher resting heart rate observed in this population.

## 1. Introduction

Respiratory sinus arrhythmia is a physiological phenomenon, which can be recognized on an electrocardiogram (ECG) or during chest auscultation. This automatic mechanism causes heart rate (HR) to increase during inspiration, enhancing effective gas exchange in the lungs, and to decrease during expiration, limiting energy expenditure on unnecessary heartbeats and preventing ineffective ventilation. The effector pathway of RSA is mediated by parasympathetic neurons of vagus nerve; thus, higher vagal tone enhances the RSA. In children and young adults, in whom the change of HR according to the respiratory phase is more pronounced, RSA can resemble heart rhythm disturbances; thus, auscultation on breath-holding helps in differentiating RSA from other arrhythmias. To date, the connection between RSA and parasympathetic activity is rarely used in clinical practice; however, the laboratory settings enable analysis of more complex patterns of heart rate variability (HRV). According to current studies, higher HRV is associated with good exercise capacity [1,2,3] and psychosocial well-being [4]. A healthy lifestyle and fitness increase HRV, while its decrease occurs in many non-communicable diseases in adults and children alike [5,6,7]. A proper analysis of HRV requires one to obtain a 5 to 10 min resting, good quality ECG recording, which is time-consuming and difficult in clinical practice. However, a simple recognition of presence of RSA or its absence can be determined during a routine physical examination. The present study was conducted to assess the presence of RSA and its magnitude in healthy children and to investigate the connection between anthropometric parameters and RSA in clinical settings.

## 2. Materials and Methods

The study was conducted as part of a yearly program aimed at early prevention of non-communicable diseases. The program was addressed to all fifth grade students from the town of Sopot (about 36,000 inhabitants) in the years 2015–2018, provided that written consent was given by their legal guardians. Out of 681 children who took part in the screening, 626 children aged 9.67 to 11.67 were included in the study. The reasons for disqualification were: improper age (*n* = 13), school absence (*n* = 39) and heart rhythm disturbances registered on the recording (*n* = 3). Health status of the participants was verified by written questionnaires addressed to their parents. Two patients with serious comorbidities (type 1 diabetes and familial hypercholesterolemia) were already excluded for the previously mentioned reasons. Intake of medications: oral supplementation of thyroid hormones (with normal TSH and fT4 levels measured within 3 days from the screening), antihistamines, leukotriene receptor antagonists, methylphenidate, as well as inhaled beta2-adrenergic agonists and steroids were not a reason for exclusion. In the case of an acute infection on the day of screening, the visit was postponed. A transthoracic echocardiography performed with GE Vivid E95 ultrasound machine comprised basic pediatric projections: subcostal view, four- and five-chamber views, long axis view and short axis at the level of aortic valve, atrioventricular valves and papillary muscles, as well as suprasternal view of the aortic arch. The protocol included measurements of left ventricular dimensions, left atrium and aortic root diameters along with the assessment of flow pattern through atrioventricular, aortic and pulmonary valves. Patent foramen ovale, small atrial septal defects without signs of hemodynamic importance, first degree mitral, tricuspid or pulmonary regurgitations, as well as a bicuspid aortic valve without dysfunction were not considered to be remarkable pathologies. None of the subjects examined according to the aforementioned protocol had signs of cardiomyopathy or substantial heart defects. Anthropometric measurements were performed according to the previously described protocol [8]. Twelve-lead, ten-second electrocardiograms were recorded by medical students after at least four years of medical school and a 2 h practical training session led by a specialist in internal medicine. The ECGs were recorded in a specially arranged, quiet room using the Mortara Eli 280 instrument. The sampling rate was 10,000 kHz, chart speed 25 mm/s and calibration 10 mm/mV. The patients were asked not to exercise or eat heavy meals within 2 h before the ECG and lay down in supine position for at least 1 min before. The technical quality of each recording was assessed straightaway by the operators, and in case it was not satisfactory, the ECG was repeated. Measurements of HR, PR, QRS and QT were taken automatically and visually revised, while measurements of RR intervals were taken semi-automatically by one person in lead II after magnifying the digital ECG image by 200% in Adobe Acrobat Reader DC software. For the quantitative analysis of sinus arrhythmia, we calculated the difference between the longest and shortest RR interval from the recording (pvRSA) as well as root mean square of successive RR differences (RMSSD), a parameter that is used in HRV analysis and has been validated for short ECG recordings as well. A previously described exponential model [9] was used to correct RMSSD for HR.

We investigated statistical differences in electrocardiographic and anthropometric parameters between rhythmic and non-rhythmic subjects. A 160 millisecond difference between the longest and shortest beat from the recording was set as the cut-off point, which is consistent with national guidelines for ECG interpretation [10]. Additionally, the indices of RSA were summarized for the following groups in accordance with national BMI standards from the OLAF study [11]:

- Normal weight—≥5th and <85th percentile,
- Underweight—<5th percentile,
- Overweight—≥85th and <95th percentile,
- Obesity—≥95th percentile.

The statistical analysis was performed using the Statistica13 software. The Shapiro–Wilk test was used to determine normal distribution of the sample and the Mann–Whitney U test was used for the comparative analysis of independent samples. Spearman rank correlation coefficients were calculated to assess correlations between analyzed variables. A *p* < 0.05 was considered statistically significant.

## 3. Results

We examined 626 students, 326 (52.1%) of which were male. The statistical analysis of anthropometry revealed no significant difference between boys and girls in age, height, weight or BMI. Waist circumference (*p* < 0.001) and systolic blood pressure (*p* = 0.037) were greater in boys as compared to girls, in contrast to HR that was significantly higher in girls (*p* < 0.001). Among the electrocardiographic variables, QRS length was greater in boys (*p* < 0.001), while QTc was greater in girls (*p* < 0.001).

In 43% of the recordings, heart rate was rhythmic, whereas in the remaining 57% we recognized respiratory sinus arrhythmia. Adolescents with RSA were characterized by a significantly lower HR (*p* < 0.001), lower weight (*p* = 0.009), BMI (*p* = 0.005) and systolic (*p* = 0.018) and diastolic (*p* = 0.004) blood pressure as well as shorter QTc (*p* < 0.001). No differences in terms of height and electrocardiographic variables were observed. Descriptive statistics of the study population are presented in Table 1.

Considering the national norms, 77.5% of children had normal body composition (BMI between the 5th and 85th pc), 5.4% were underweight, 13.1% were overweight and 4.0% had developed obesity. The indices of RSA in distinct groups are summarized in Table 2.

Despite differences in average HR, no substantial differences regarding pvRSA and RMSSD between boys and girls were observed, whereas RMSSDc was significantly higher in girls (*p* = 0.046). In the comparison of subjects with BMI above 85th percentile to the normal-weight group (Table 2), subjects with high BMI were characterized by significantly higher HR (*p* < 0.001), whereas the RSA indices were significantly higher in the normal-weight population (*p* < 0.001 for pvRSA and RMSSD; see Figures S1 and S2). RMSSDc was not affected by excessive body mass (Figure S3) either in the whole group or in the sex-specific calculations. There were important inverse correlations of heart rate with pvRSA (r = −0.52) (Figure S4) and RMSSD (r = −0.58) (Figure S5).

The Spearman rank correlation revealed no important connections between the indices of RSA and students’ anthropometry (r < 0.20) (Figure S6).

## 4. Discussion

The study showed that children with respiratory sinus arrhythmia were thinner and had lower blood pressure compared to their peers with rhythmic HR. However, considering the inverse correlation between HR and RSA, together with the tendency to higher HR among overweight children, it should be assumed that anthropometric parameters have no direct influence on RSA in 11-year-olds. Noticeably, despite considerable dispersion of RSA among the students, none of them suffered from a structural heart disease.

The close relationship between HR and the grade of RSA is understandable from both a physiological and mathematical approach. Firstly, it is the vagus nerve that is responsible for slowing down the HR and increasing its variability as well. Secondly, lower HR is directly associated with longer interbeat intervals, which permits greater absolute differences between them. This connection has been reported previously [12,13] and leads to developing HRV-correcting formulas according to prevailing HR [14].

Interestingly, RMSSDc differed substantially between boys and girls, whereas no other indices of RSA did. This finding is a result of higher heart rate in girls which masked differences in RSA. Since prevalence of RSA in relation to sex was not a subject of the present study, we leave it for further investigation.

The association of elevated HR with overweight and obesity in children has been described by previous authors [15,16,17,18,19]. Hirsh et al. [20] found that 10% weight gain was connected to an increase in HR and a loss of its variability, which is in line with our results. Thus, it can be assumed that HR and HRV in the overweight remain under the control of the same physiological mechanism. Mathematical adjustment of HRV with HR affects the results and their interpretation considerably [14]. In groups that are not age-homogenous, correction for HR may lead to contrary conclusions (HRV decreases with age) [17].

It should be emphasized that 10 s ECG recording is not a reference method for the measurement of HRV. Considering the multitude of factors that have an effect on HR and its variability as well as numerous components of the overall HRV (from short to ultra-long term variability), a study on HRV requires optimal conditions and quality equipment. The aim of our study was to analyze only one component of HRV: RSA, in a very short recording, so the methodology did not meet the standards for HRV analysis. Nevertheless, the data on RSA in children and adolescents in the literature are mainly extracted from 5 min HRV analysis, with only a few studies on short recordings [21,22]. To our knowledge, this has been the first study on a large group of children that focuses on implications of RSA in the short ECG. Moreover, the effect of excessive body mass on HRV remains uncertain. A number of studies proved lower HRV in children with obesity [16,18,23]; however, a study by Kaufman et al., conducted on a smaller group of children [24], only revealed the difference between normal-weight and obese subjects, in contrast to the overweight. Moreover, Mazurak et al. [25], who analyzed the effect of weight reduction on HRV, reported no difference between obese and normal-weight adolescents in the initial examination and a substantial HRV increase in the subjects with obesity after dietary intervention. This incoherence might be attributed to differences in participants’ age and applied methodology, including time and length of the recording, patient’s preparation and analyzed HRV metrics in different studies. Another hypothesis, which renders the issue of HRV regulation even more complex, is that longer obesity duration leads to adaptative changes in the autonomic nervous system, and therefore, causes less dysregulation than its initial phase [19]. In our study, we observed lower HRV indices in subjects with a BMI above 85 pc (overweight and obesity). However, the medium values for the subjects with obesity were higher than those of the overweight ones. Considering the disproportion between the “obesity” and “normal-weight” groups (25 vs. 485 children), we leave this subject for further evaluation in future studies.

In terms of study limitations, it should be mentioned that the parasympathetic activity is a result of multiple factors that were not considered in the present research. HRV is not stable across time: not only is it age-dependent, but it also displays a certain circadian rhythm. In general, greater values of HRV parameters are observed in the morning than in the evening [26]. In our study the maximal age difference between students was two years and all the ECGs were recorded between 8 a.m. and 12 to minimize intragroup variation; however, RSA can be temporarily disrupted by momentary sympathetic activation secondary to emotional factors. On account of the above, we created similar settings for every examination and provided each patient with the same information before the procedure. In some of the previous studies, participants were shown the same movie to eliminate the situational differences. However, one’s emotional state is not only shaped by external circumstances, but also by one’s personality and an individual response to visual and sonic stimuli, which is difficult to predict or influence. This sort of bias cannot be excluded in a daytime analysis of RSA, or HRV in a wider sense. There were some attempts to measure the night-time HRV during certain phases of sleep, which led to promising results [17]. To our knowledge, the relationship between HRV and BMI in pediatric populations during sleep has not yet been evaluated.

Using the 10 s recordings for the sake of the present study can be considered its limitation and a strength as well. Admittedly, the smaller number of RR intervals in the analyzed dataset is more prone to bias and has lower reproducibility in comparison with larger ones. However, in reference to the aim of the study, which was analysis of RSA in clinical settings, we assume that the method was adequate, since it corresponds better with RSA assessment during an outpatient medical visit.

The choice of RSA indices was adjusted to the applied methodology as well. Even though multiple parameters, based on either RR interval duration or frequency, are used to describe HRV, only a few are possible to derive from a small dataset. RMSSD not only was proven to correspond with the parasympathetic activity [26], but also was standardized on a large number of short ECGs [9]. The aforementioned study of van den Berg et al. included the standard deviation of RR intervals (SDNN) as well. However, this parameter is strongly affected by the analyzed number of intervals, which in our study varied across the participants according to their heart rate (10 intervals for 60 bpm and 15 for 90 bpm). Additionally, the SDNN was found to remain under both parasympathetic and sympathetic influence [2]. Despite the absence of reference values for pvRSA dedicated to short recordings, we chose it for its simplicity and satisfactory correspondence with other indices.

## 5. Conclusions

The presence and magnitude of RSA in children assessed during medical screening has no clear implications regarding their anthropometry. Considering an inverse correlation between indices of RSA with HR together with noticeable diversification of resting HR among pediatric patients, the analysis of RSA should include correction for HR.

## Figures and Tables

**Table 1:** Descriptive statistics of the study population.

	Total		Boys		Girls		Rhythmic		Non-Rhythmic	
N (%)	626 (100%)		326 (52.1%)		300 (47.9%)		269 (43.0%)		357 (57.0%)	
	mean	SD	mean	SD	Mean	SD	mean	SD	mean	SD
Age [years]	10.77	0.50	10.78	0.48	10.75	0.48	10.81	0.49	10.74	0.50
Height [cm]	147.44	7.28	147.34	7.21	147.55	7.36	147.70	7.33	147.24	7.24
Weight [kg]	39.59	8.98	40.03	9.54	39.11	8.33	40.58	9.11	38.84	8.83 *
BMI [kg/m^2^]	18.07	3.07	18.27	3.19	17.86	2.93	18.46	3.16	17.78	2.98 *
Wc [cm]	64.90	8.61	66.15	9.20	63.55	7.72 **	65.87	8.80	64.18	8.41 *
Hc [cm]	77.67	8.07	78.12	8.25	77.19	7.87	78.71	8.10	76.90	7.97 *
SBP [mmHg]	106.52	9.73	105.77	9.66	107.35	9.75 *	107.50	9.80	105.79	9.62 *
DBP [mmHg]	63.90	8.02	63.45	7.75	64.39	8.29	64.93	8.10	63.12	7.87 *
HR [bpm]	80.99	13.71	78.44	12.85	83.76	14.09 **	87.70	14.44	75.94	10.65 **
PQ [ms]	135.69	17.84	136.65	18.49	134.64	17.08	135.63	18.53	135.74	17.33
QRS [ms]	86.96	9.20	88.48	9.55	85.30	8.51 **	86.70	9.24	87.15	9.18
QRS axis [°]	66.15	26.65	63.39	26.91	69.16	26.09	64.79	27.94	67.19	25.63
QTc [ms]	411.32	21.08	408.02	20.81	414.91	20.81 **	418.94	20.35	405.58	19.78 **

* indicates a statistically significant difference between subgroups (*p* < 0.05); ** *p* < 0.001; BMI—body mass index, Wc—waist circumference, Hc—hip circumference, SBP—systolic blood pressure, DBP—diastolic blood pressure, HR—heart rate.

**Table 2:** HRV metrics in distinct groups in regards to sex and stature. Data presented as mean (SD).

	N (%)	HR	pvRSA	RMSSD	RMSSDc
total	626 (100%)	80.99 (13.71) *	203.30 (110.55) **	53.92 (34.53) **	99.91 (51.41) **
boys	326 (52.1%)	78.44 (12.85) *	210.78 (113.98) *	56.58 (35.55) *	**96.05 (48.78)**
girls	300 (47.9%)	**83.76 (14.09)**	195.18 (106.30) *	51.02 (33.19) *	**104.10 (53.90)**
normal weight	485 (77.5%)	80.37 (13.68)	210.46 (113.23)	55.77 (35.18)	101.61 (51.55)
overweight	82 (13.1%)	84.58	159.41 (84.26)	40.99 (25.80)	88.64 (52.91)
obesity	25 (4.0%)	86.52 (15.67)	186.62 (108.08)	53.73 (39.65)	104.47 (46.20)
underweight	34 (5.4%)	81.21 (13.91)	219.31 (107.24)	58.88 (34.11)	99.37 (47.86)

Parameters that differed between boys and girls are marked in bold; * = substantial difference (*p* < 0.05) between normal-weight children and subjects with BMI ≥ 85 pc; ** = *p* < 0.001; pvRSA—the difference between the longest and the shortest RR interval (RRmax-RRmin); RMSSD—root mean square of differences between successive RR intervals; RMSSDc—RMSSD corrected for heart rate.

## Data Availability

The data presented in this study are available on request from the corresponding author. The data are not publicly available due to privacy restrictions.

## Supplementary Materials

The following are available online at https://www.mdpi.com/article/10.3390/ijerph19010566/s1, Figure S1: A box-plot showing pvRSA in subjects with normal-weight students (5–85 pc of BMI) compared to the group with overweight and obesity (BMI > 85 pc), Figure S2: A box-plot showing RMSSD in subjects with normal-weight students (5–85 pc of BMI) compared to the group with overweight and obesity (BMI > 85 pc), Figure S3: A box-plot showing RMSSDc in subjects with normal-weight students (5–85 pc of BMI) compared to the group with overweight and obesity (BMI > 85 pc), Figure S4: A scatter chart showing interdependence between heart rate and pvRSA, Figure S5: A scatter chart showing interdependence between heart rate and RMSSD, Figure S6: A scatter chart showing interdependence between heart rate and RMSSDc.
