# Supplementary material for: Anthropometric Determinants of Respiratory Sinus Arrhythmia in Children
Source: Int J Environ Res Public Health. 2022 Jan 5;19(1):566. doi: 10.3390/ijerph19010566 (PMC8744931; doi:10.3390/ijerph19010566)
Supplement: Supplementary file 1 [file ijerph-19-00566-s001.zip › ijerph-1458448-supplementary.pdf]

International Journal of
Environmental Research and Public Health

MDPI

Article

Anthropometric Determinants of Respiratory Sinus Arrhythmia in Children

Paulina Lubocka [1,*], Robert Sabiniewicz [1], Klaudia Suligowska [2,3] and Tomasz Zdrojewski [2]

1 Department of Pediatric Cardiology and Congenital Heart Disease, Medical University of Gdańsk, Dębinki Street 7, 80-952 Gdańsk, Poland; sabini@gumed.edu.pl
2 Department of Preventive Medicine and Education, Medical University of Gdańsk, Dębinki Street 7, 80-952 Gdańsk, Poland; klaudia.suligowska@gumed.edu.pl (K.S.); tz@gumed.edu.pl (T.Z.)
3 Department of Dental Techniques and Masticatory System Dysfunctions, Medical University of Gdańsk, Tuwima Street 15, 80-210 Gdańsk, Poland
* Correspondence: paulinalubocka@gumed.edu.pl

Citation: Lubocka, P.; Sabiniewicz, R.; Suligowska, K.; Zdrojewski, T. Anthropometric Determinants of Respiratory Sinus Arrhythmia in Children. *Int. J. Environ. Res. Public Health* **2022**, *19*, 566. https://doi.org/10.3390/ijerph19010566

Academic Editor: Bożena Werner

Received: 26 October 2021
Accepted: 31 December 2021
Published: 5 January 2022

Publisher's Note: MDPI stays neutral with regard to jurisdictional claims in published maps and institutional affiliations.

Copyright: © 2022 by the authors. Licensee MDPI, Basel, Switzerland. This article is an open access article distributed under the terms and conditions of the Creative Commons Attribution (CC BY) license (http://creativecommons.org/licenses/by/4.0/).

Supplementary materials

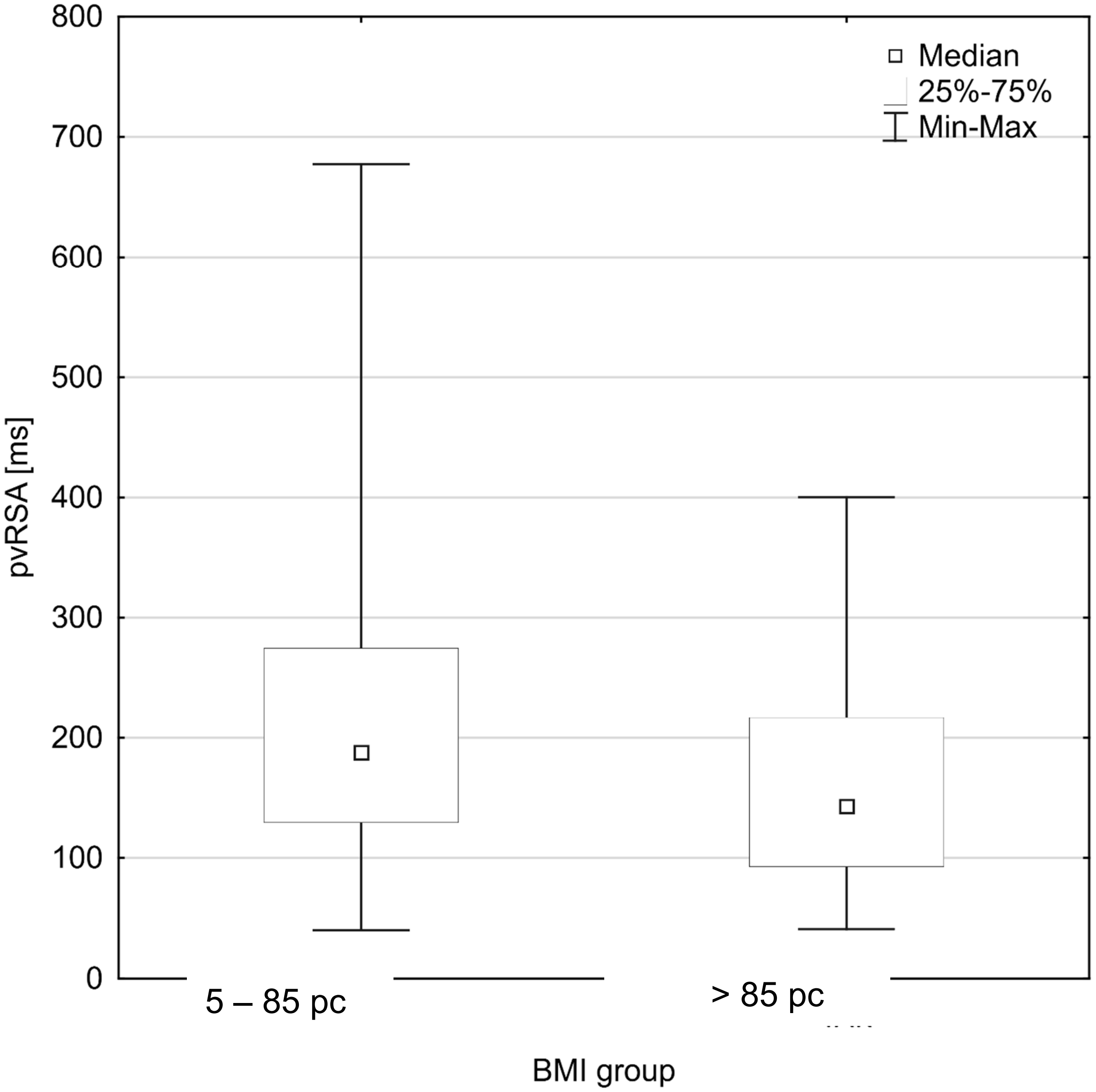

Figure S1. A box-plot showing pvRSA in subjects with normal-weight students (5–85 pc of BMI) compared to the group with overweight and obesity (BMI > 85 pc). The difference was statistically significant ($p < 0.001$).

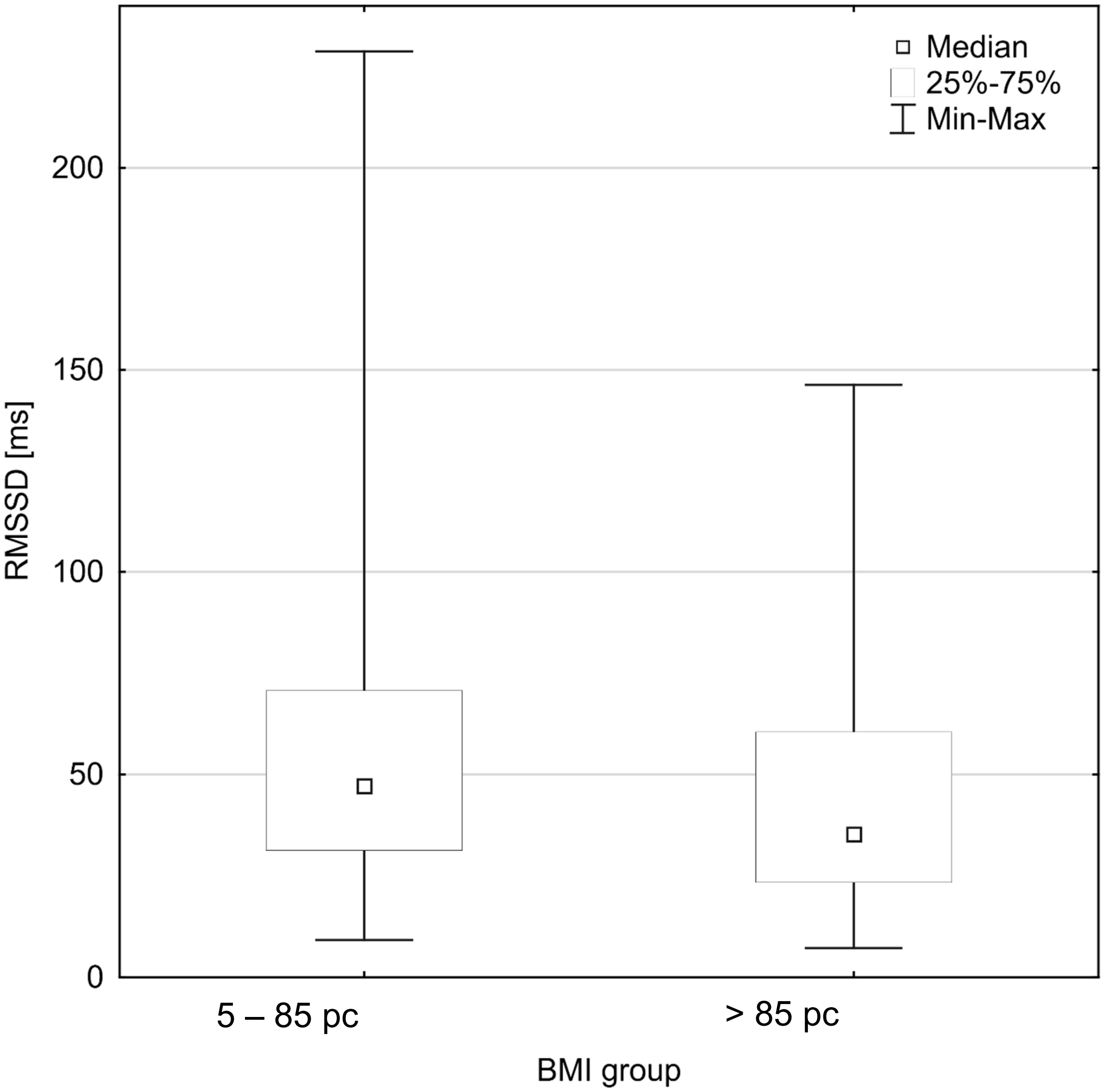

Figure S2. A box-plot showing RMSSD in subjects with normal-weight students (5–85 pc of BMI) compared to the group with overweight and obesity (BMI > 85 pc). The difference was statistically significant ($p < 0.001$).

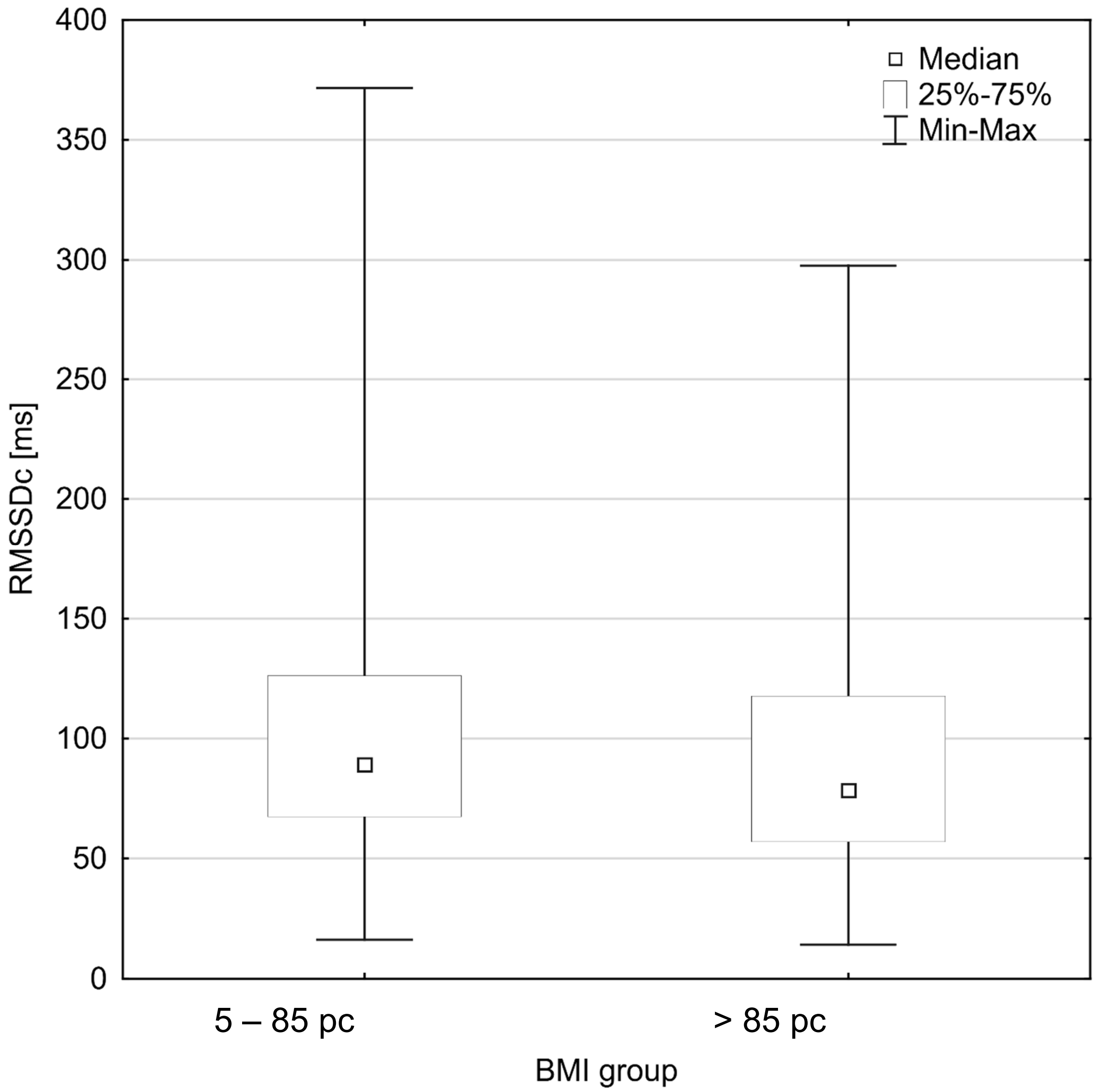

Figure S3. A box-plot showing RMSSDc in subjects with normal-weight students (5–85 pc of BMI) compared to the group with overweight and obesity (BMI > 85 pc). No substantial difference between the groups was reported ($p = 0.059$).

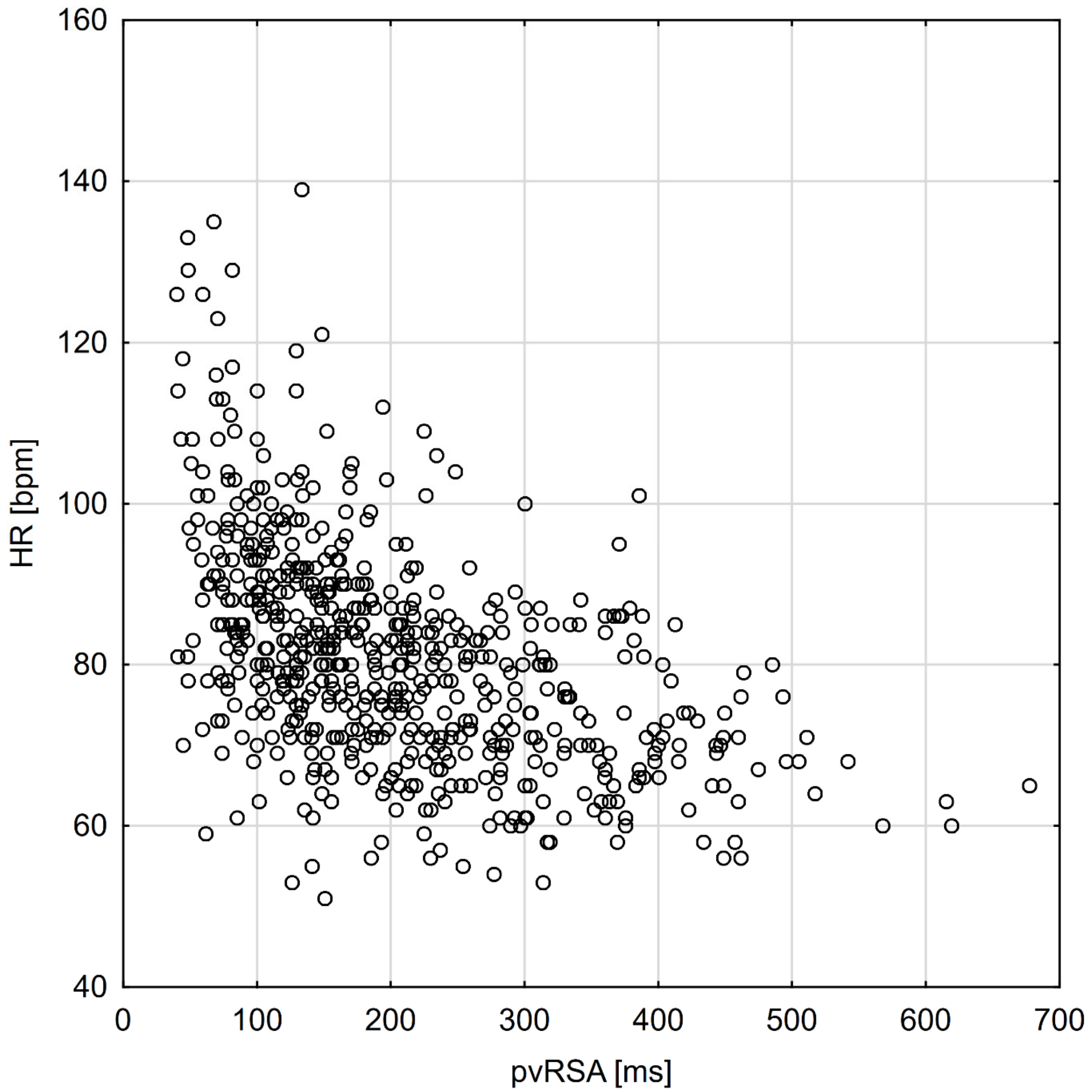

Figure S4. A scatter chart showing interdependence between heart rate and pvRSA. A substantial correlation was observed ($r = -0.52$).

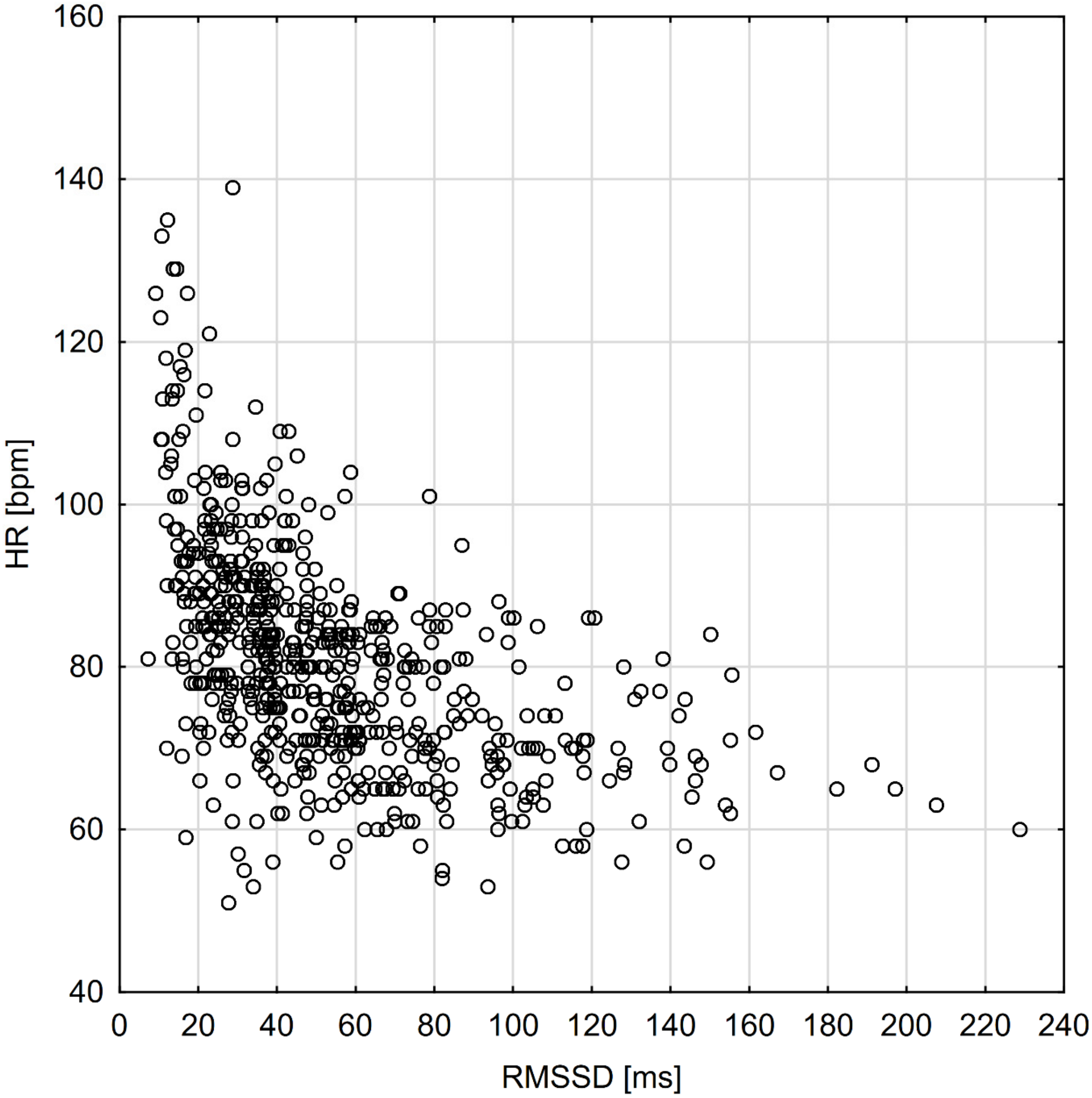

Figure S5. A scatter chart showing interdependence between heart rate and RMSSD. A substantial correlation was observed (r = −0.58).

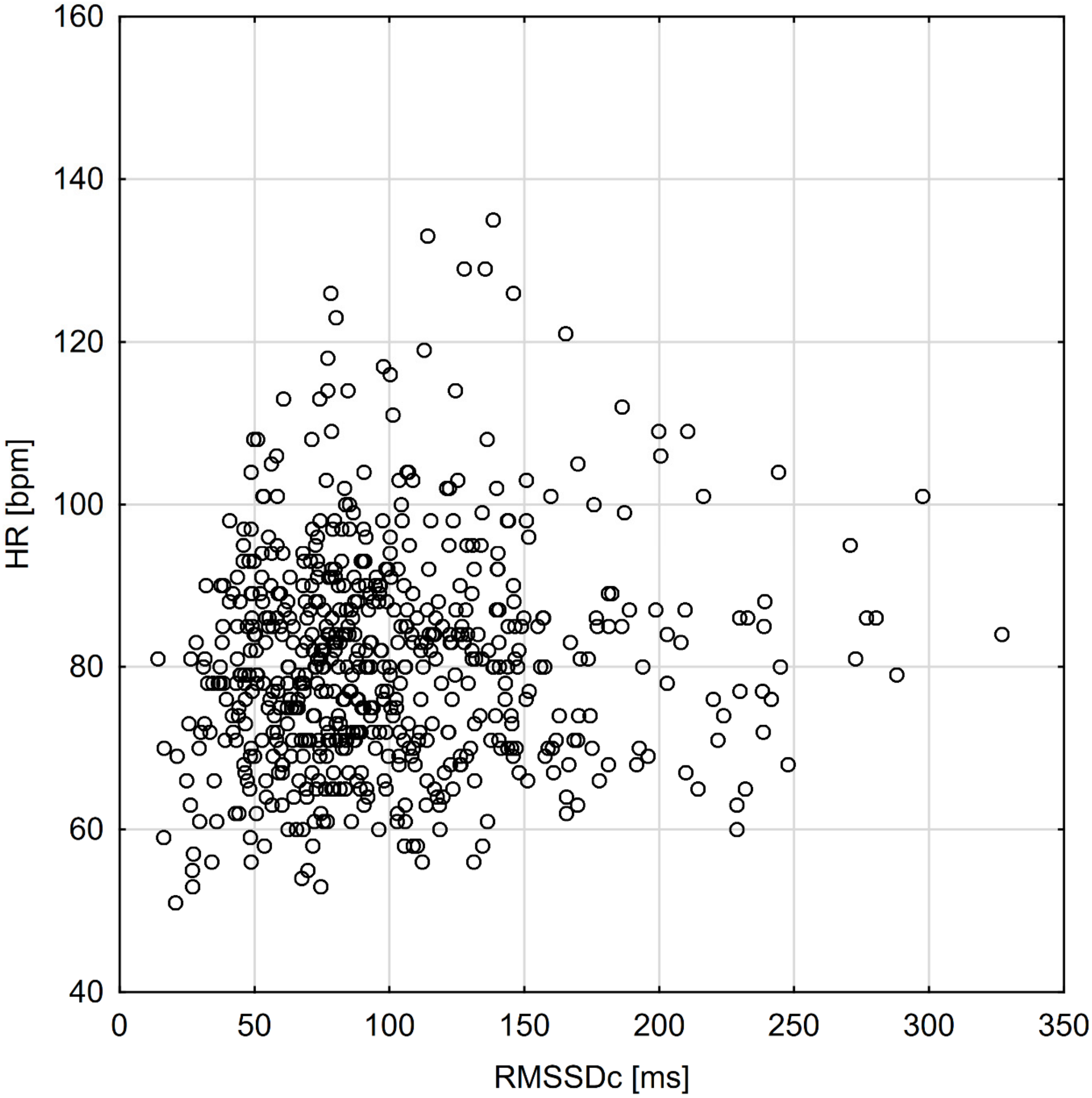

Figure S6. A scatter chart showing interdependence between heart rate and RMSSDc. No important correlation was observed between the variables.
